# Pluripotent stem cells secrete Activin A to improve their epiblast competency after injection into recipient embryos

**DOI:** 10.1007/s13238-017-0470-y

**Published:** 2017-10-12

**Authors:** Jinzhu Xiang, Suying Cao, Liang Zhong, Hanning Wang, Yangli Pei, Qingqing Wei, Bingqiang Wen, Haiyuan Mu, Shaopeng Zhang, Liang Yue, Genhua Yue, Bing Lim, Jianyong Han

**Affiliations:** 1 State Key Laboratory for Agrobiotechnology, College of Biological Sciences, China Agricultural University, Beijing, 100193 China; 2 Animal Science and Technology College, Beijing University of Agriculture, Beijing, 102206 China; 3 Temasek Life Sciences Laboratory, National University of Singapore, Singapore, 117604 Singapore; 4 Stem Cell and Developmental Biology, Genome Institute of Singapore, Singapore, 138672 Singapore; 5 Beijing Advanced Innovation Center for Food Nutrition and Human Health, China Agricultural University, Beijing, 100193 China; 6 State Key Laboratory of Animal Nutrition, Institute of Animal Sciences, Chinese Academy of Agricultural Sciences, Beijing, 100193 China

**Keywords:** pluripotent stem cells, 4-cell embryo injection, secreted proteins, Activin A, chimeric mice

## Abstract

It is not fully clear why there is a higher contribution of pluripotent stem cells (PSCs) to the chimera produced by injection of PSCs into 4-cell or 8-cell stage embryos compared with blastocyst injection. Here, we show that not only embryonic stem cells (ESCs) but also induced pluripotent stem cells (iPSCs) can generate F0 nearly 100% donor cell-derived mice by 4-cell stage embryo injection, and the approach has a “dose effect”. Through an analysis of the PSC-secreted proteins, Activin A was found to impede epiblast (EPI) lineage development while promoting trophectoderm (TE) differentiation, resulting in replacement of the EPI lineage of host embryos with PSCs. Interestingly, the injection of ESCs into blastocysts cultured with Activin A (cultured from 4-cell stage to early blastocyst at E3.5) could increase the contribution of ESCs to the chimera. The results indicated that PSCs secrete protein Activin A to improve their EPI competency after injection into recipient embryos through influencing the development of mouse early embryos. This result is useful for optimizing the chimera production system and for a deep understanding of PSCs effects on early embryo development.

**Electronic supplementary material:**

The online version of this article (doi:10.1007/s13238-017-0470-y) contains supplementary material, which is available to authorized users.

## INTRODUCTION

A conventional and useful approach for understanding gene function has involved producing genetically modified mice from embryonic stem cells (ESCs) that contain genetic changes since the successful derivation of mouse ESCs from blastocysts (D; Martin, 1981; Thomas and Capecchi, 1987). ESC-based transgenic mice are usually produced by the introduction of ESCs into diploid host embryos, generally blastocysts (Ramirezsolis et al., 1993; Stewart, 1993), resulting in chimeric mice that are only partially generated from ESCs. It takes a lot of time to produce homozygous mutant mice that are suitable for phenotyping. Previous reports have shown that the injection of ESCs into 4- or 8-cell stage embryos produces F0 nearly 100% ESC-derived mice (ES-mice), with full germline transmission that permits immediate phenotypic analysis (Huang et al., 2008; Poueymirou et al., 2007). These methods significantly accelerated the process of gene function research.

Normal diploid embryos contribute to placental development and participate in fetal development (Rossant and Tam, 2009; Zernicka-Goetz et al., 2009). Injections of ESCs into diploid embryos could theoretically generate chimeric mice that are derived from both ESCs and host embryos. However, this outcome is not applicable to the injection of ESCs into the 4- or 8-cell embryos that can produce the ES-mice. The host embryo does not contribute to the fetal development in this method, which is similar to the tetraploid embryo that only contributes to extraembryonic tissues, such as the placenta (Eakin and Behringer, 2003). These results demonstrate that exogenous ESCs injected into 4- or 8-cell stage embryos have an impact on the host embryo developmental fate. Mouse ESCs injected into 8-cell stage embryos modified the pattern of cell lineage specification (Humiecka et al., 2016), which supports this view. However, it has not yet been completely elucidated how donor ESCs regulate the embryo developmental fate. Recent reports have indicated that ESCs or differentiating ESC-secreted proteins may affect the growth and development of exogenous cells, such as cell migration and myogenesis (Ngangan et al., 2014; Yousef et al., 2014). Therefore, we hypothesized that ESC-secreted proteins may affect the embryo developmental fate following the injection of ESCs into embryos.

Studies have shown that induced pluripotent stem cells (iPSCs) resemble ESCs in pluripotency, morphology, and differentiation abilities (Takahashi et al., 2007; Takahashi and Yamanaka, 2006). Notably, iPSCs could produce not only chimeric mice by blastocyst injection but also full-term offspring by tetraploid complementation (Kang et al., 2011; Maherali et al., 2007; Wernig et al., 2007; Zhao et al., 2009). Nevertheless, it remains unclear whether F0 nearly 100% iPSC-derived mice (iPS-mice) can be efficiently produced from 4- or 8-cell embryo injection.

In this work, we revealed that both ESCs and iPSCs could generate F0 nearly 100% donor cell-derived mice by the injection of cells into 4-cell stage embryos, and the 4-cell stage embryo injection assay had a “dose effect”. In comparison to the injection of 20 ESCs, more coat chimeras were produced by the injection of fewer ESCs (i.e., 10 ESCs) into embryos. In addition, mouse ESC and iPSC-secreted protein Activin A was found to stunt epiblast (EPI) lineage development and to stimulate the development of the trophectoderm (TE) lineage in host embryos. We also showed that using embryos cultured with Activin A as the host increases the contribution of ESCs to the chimeras produced by conventional blastocyst injection. This process may explain the phenomenon that the injection of ESCs or iPSCs into 4-cell stage embryos yields F0 generation mice with a greater ESC or iPSC contribution than does blastocyst injection. The results indicated that PSCs secrete protein Activin A to improve their EPI competency after injection into recipient embryos via impacting on the development of mouse early embryos.

## RESULTS

### An increase in the number of cells injected into 4-cell stage embryos efficiently produces F0 generation ES-mice or iPS-mice

**Figure 1:**
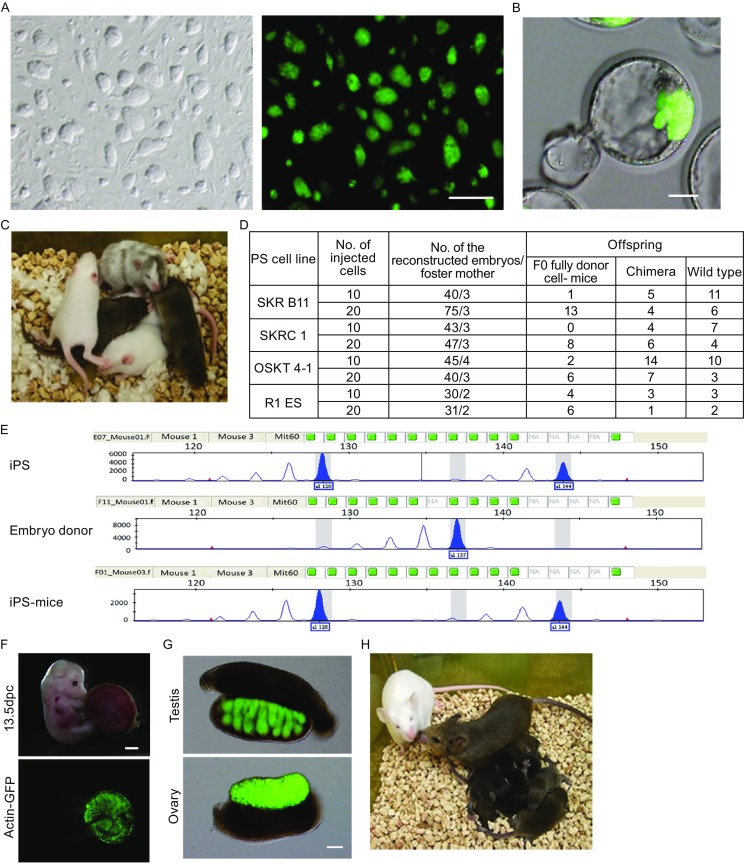
**Generation of F0 iPS-mice by 4-cell stage embryo injection**. (A) Mouse iPSC colonies (left panel, bright field; right panel, Oct4-GFP). Scale bar, 100 μm. (B) Blastocyst from 4-cell embryo injection. Scale bar, 20 μm. (C) F0 generation mice produced by 4 cell-stage embryo injection. (D) Summary of the generation of F0 fully iPSC or ESC-derived mice by 4-cell embryo injection of 20 or 10 donor cells. See also Fig. S1. (E) Representative microsatellite analysis of tails from iPSC-derived mice using D12Mit60 primers. See also Table S1. (F) Chimeric embryo at E13.5 produced by 4-cell stage embryo injection. Scale bar, 1 mm. (G) Gonads of E13.5 embryos from 4-cell embryo injection. Scale bar, 200 μm. (H) Representative F0 iPSC-derived mouse (agouti color) and offspring

In previous studies, we generated mouse iPS cell lines by different combinations of factors (Oct4, Sox2, Klf4 and Tbx3, termed OSKT; Sox2, Klf4 and Nr5a2, termed SKR; Sox2, Klf4, Nr5a2 and c-Myc, termed SKRC) that were morphologically similar to mouse ESCs, were Oct4-GFP positive (Fig. 1A), could produce chimeras, and could even produce iPS-mice via tetraploid complementation assay (Han et al., 2010; Heng et al., 2010). We hypothesized that these cells could produce iPS-mice by injecting approximately 10 cells (9–11 cells) into 4-cell stage embryos based on previous reports (Huang et al., 2008; Poueymirou et al., 2007). While the injected embryos developed into blastocysts, these iPSCs could contribute to the inner cell mass (ICM) (Fig. 1B). The live mice produced by the 4-cell method were divided into three types: iPS-mice, chimeras, and host-derived mice (Fig. 1C). However, only a fewer iPS-mice were produced compared with wild-type R1 ESCs (Fig. 1D). To test whether the iPS-mice were chimeras, we randomly selected 2 and collected their tissues, such as heart, liver, spleen, lung and brain, to extract genomic DNA. We then used D12Mit60 primers to perform a microsatellite assay. Microsatellite analysis revealed that the iPS-mice generated by 4-cell embryo injection were all from iPSCs rather than host embryos (Fig. 1E and Table S1). To further detect the fate of iPSCs following injection into 4-cell stage embryos, we injected Oct4-GFP iPSCs into the embryos carrying actin-GFP. The fluorescence location showed that Actin-GFP was negative in the fetus but positive in the placenta at embryonic day (E) 13.5. This result also demonstrates that iPSCs rather than host embryo EPI contributed to the fetus (Fig. 1F). An analysis of the gonads of high chimeric embryos further showed a high contribution of iPSCs with GFP expression to male and female gonads at E13.5, indicating that 4-cell stage embryo injection resulted in a high iPSC contribution to the gonad (Fig. 1G). We further identified that the adult iPS-mice exhibited 100% germline transmission (Fig. 1H).

Interestingly, while increasing the injection cell numbers to approximately 20, we found that these cells could efficiently produce ES-mice or iPS-mice, with coats and genotypes that were the same as the pluripotent stem cell background (Fig. 1C and 1D). This result suggests that the 4-cell stage embryo injection assay had a “dose effect” and that the injected pluripotent stem cells might force the host embryo blastomeres to change their fates, with the exogenous cells replacing the EPI to develop into a fetus. To confirm the conception, we used the G4-DsRed-MST ESCs (Vintersten et al., 2004) for the 4-cell embryonic injection assay (Fig. S1A and S1B). In addition to the evaluation of *in vivo* developmental potential, we performed immunofluorescent staining for the aggregation embryos at E4.5 to check Nanog localization in the ICM (Fig. S1A). In mouse embryo development, Nanog specifically expresses in the EPI, which gives rise to the future fetus, so Nanog staining can display the EPI cells (Rossant and Tam, 2009; Zernicka-Goetz et al., 2009). Immunofluorescent staining showed that EPI cells (defined on the basis of Nanog expression) were completely developed from ESCs in some blastocysts (Fig. S1C and S1D). As the numbers of injected ESCs increased, the percentage of blastocysts, whose EPI cells were only from ESCs, also increased. Of the blastocysts, 75% (ESCs-derived EPI) were generated by the injection of 20 cells, and 31.25% of the blastocysts (ESCs -derived EPI) were derived by the injection of 10 cells (Fig. S1D). This result is consistent with the fact that F0 nearly 100% ESC and iPSC-derived mice can be produced by 4-cell stage embryo injection. This also suggests that donor ESCs impede the EPI lineage development of host embryos.

### ESC and iPSC secretions hinder EPI lineage development

**Figure 2:**
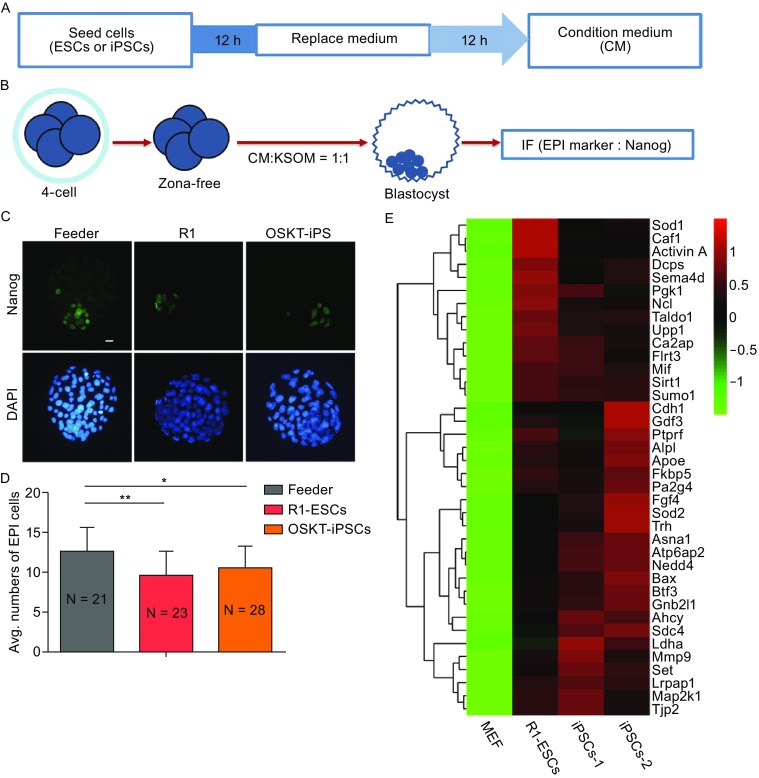
**Secretions from ESCs and iPSCs affect EPI development**. (A) Schematic of the method used to collect the condition medium. (B) Experimental design. Zona-free embryos at 4-cell stage were treated in the mixed medium containing KSOM and CM and then immunostained at E4.5 to test the effect of the condition medium on early embryo development fate. CM, condition medium. (C) Nanog immunostaining in E4.5 embryos treated with condition medium from feeder, R1 ESCs and iPSCs. Nuclei were stained with DAPI (Blue). Scale bars, 20 μm. (D) Average numbers of EPI cells (Nanog-positive cells) in condition medium-treated embryos at E4.5. Error bars indicate SD. *P < 0.05; **P < 0.01 by ANOVA. N is the number of embryos examined. (E) Heatmap of ESC and iPSC-secreted proteins at high expression levels. The heatmap was plotted with relative protein expression

Cells can interact with each other through secreted factors. Many reports have shown that ESCs secrete cytokines and proteins that can affect the fate of other cells around them (Ngangan et al., 2014; Yousef et al., 2014). Therefore, the secretions of ESCs and iPSCs, which were injected into the 4-cell stage embryos, might hinder the EPI lineage specification during further development. To confirm this hypothesis, we chose the ESC and iPSC lines, which can produce ES-mice or iPS-mice, to collect the condition medium and to explore their effects on the EPI development of preimplantation embryos after culture *in vitro* (Fig. 2A). Zona-free embryos at the 4-cell stage were cultured in the mixed medium containing the condition medium and KSOM (1:1) (Fig. 2B). When 4-cell embryos in the mixed medium developed into E4.5 blastocysts, cell numbers of the EPI lineage (Nanog-positive cells) were detected by immunofluorescent staining. ESCs and iPSCs were maintained on feeder cells, so condition medium collected from feeder cells only was used as the control group. The results showed that a decline in the Nanog expression level was apparent (Fig. 2C), and that the EPI cell numbers were significantly reduced (Fig. 2D) in the blastocysts treated by the mixed medium, including KSOM and the condition medium from the R1 ESCs or iPSCs. These results indicate that ESC and iPSC secretions indeed suppress EPI lineage development.

### ESC and iPSC-secreted protein Activin A impedes the development of EPI lineage

**Figure 3:**
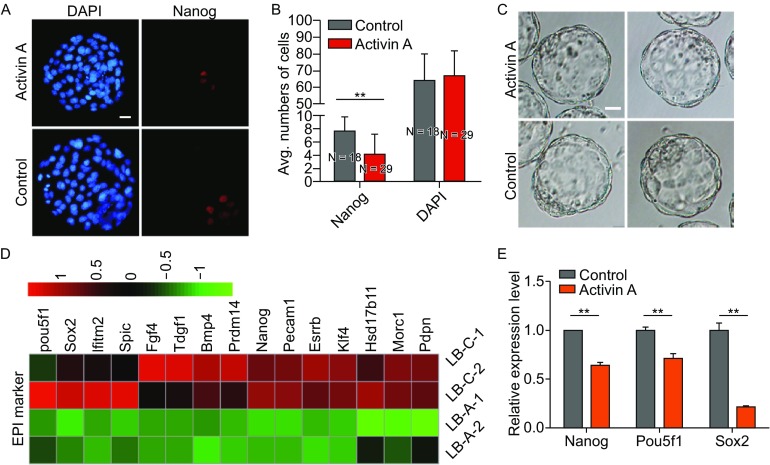
**Activin A represses EPI lineage**. (A) Nanog immunostaining in Activin A-treated and untreated embryos at E4.5. Nuclei were stained with DAPI (Blue). Scale bars, 20 μm. (B) Average numbers of EPI cells (Nanog-positive cells) in Activin A-treated and untreated embryos at E4.5. Error bars indicate SD. **P < 0.01 by ANOVA. N is the number of embryos examined. (C) Late blastocysts in the Activin A-treated group and the control group. Scale bars, 20 μm. (D) Heatmap of selected genes associated with EPI lineage. Heatmap was plotted with Z-score normalized RPKM. (E) Quantitative RT-PCR analyzed the expression of genes selected by heatmap of EPI Markers. Error bars indicate SD (*n* = 3). **P < 0.01 by Student’s *t*-test

To test the components of the condition medium, we performed mass spectrometry and then obtained a list of candidate proteins (Fig. 2E). After screening, we found that Nanog expression significantly declined and EPI cell numbers decreased in Activin A-treated embryos when its concentration was 500 ng/mL (Fig. 3A and 3B), indicating that Activin A works as a member of secreted proteins during EPI development similar to that in the condition medium. As its concentration was reduced to 100 ng/mL, the effect abated. By contrast, the effect was strengthened but not obvious as the concentration increased up to 3,000 ng/mL (data not shown). Hence, Activin A at a concentration of 500 ng/mL was used for subsequent experiments.

Activin A is a member of the transforming growth factor β (TGFβ) superfamily. Activin A exerts biological effects by interacting with its receptors, including ACVRIIA, ACVRIIB, ALK4, and ALK7 (Pauklin and Vallier, 2015). We found that the expression of Activin A receptors were higher in 4-cell embryos than in blastocysts (Fig. S2) by analyzing the published data (Fan et al., 2015). SB431542 is a potent and specific inhibitor of transforming growth factor-superfamily type I Activin receptor-like kinase (ALK) receptors such as ALK4, ALK5, and ALK7 (Inman et al., 2002). To determine whether the inhibitor affects EPI development, 4-cell embryos were cultured in the medium containing 10 μmol/L SB431542 until they developed into late blastocysts. EPI cell numbers were detected by immunofluorescent staining. The Nanog-expressing cells were increased compared with the control group, showing that more EPI cells appeared in the SB431542 group (Fig. S3A and S3B). This result is in agreement with the previous report (Ghimire et al., 2015) and further proves the effect of Activin A on early embryo development.

Next, to further confirm the impact of Activin A on early embryo development, we analyzed the transcriptomes of embryos treated with Activin A (Fig. 3C) and compared them with the control group. The analysis results showed that the expression levels of EPI marker genes such as Pou5f1, Nanog, and Sox2 were decreased in embryos in the Activin A group (Fig. 3D), which was corroborated by the quantitative RT-PCR assay (Fig. 3E). These results indicate that Activin A hinders the development of the EPI lineage.

**Figure 4:**
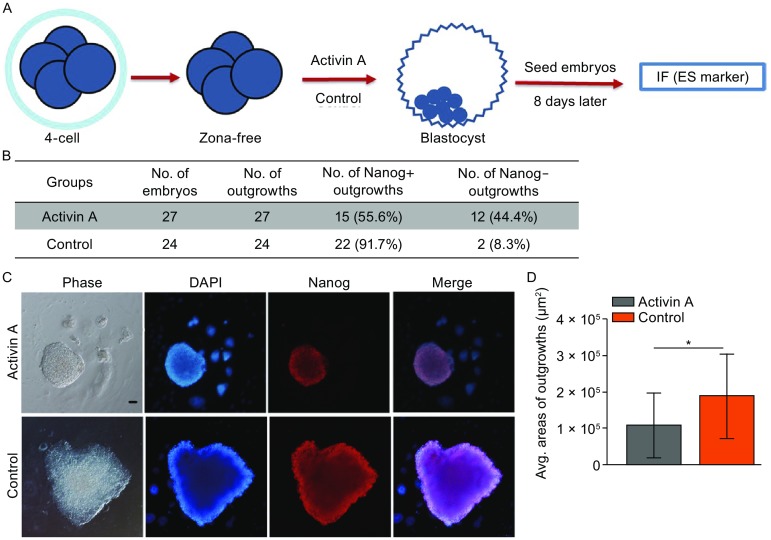
**Activin A has a negative effect on the derivation of ESCs**. (A) Experimental design for outgrowth analysis. Activin A treated-embryos were seeded on the feeder cells at E4.5 and then immunostained 8 days later to test whether Acticin A has effects on ES cell isolation. (B) Summary of outgrowths derived from Activin A treated and untreated late blastocysts. *P* < 0.05 by Chi-square test. (C) Nanog immunostaining in outgrowths derived from Activin A treated and untreated blastocysts. Nuclei were stained with DAPI (Blue). Scale bars, 50 μm. (D) Average areas (μm^2^) of Nanog positive outgrowths. Error bars indicate SD. *P < 0.05 by ANOVA

ESCs were derived from the EPI lineage in blastocysts (Evans and Kaufman, 1981; Martin, 1981). Activin A reduced the numbers of EPI cells as shown above, so we wonder whether Activin A affects the success rate of ESC isolation. Zona-free 4-cell embryos were cultured in 500 ng/mL Activin A until E4.5 and then seeded on feeder cells containing mouse ESCs medium plus PD0325901 and CHIR99021 (2i) (Fig. 4A). Eight days later, we examined numbers and areas of Nanog-positive outgrowths (Fig. 4A). Interestingly, the ratio of Nanog-positive outgrowths in the Activin A treatment group was significantly lower (Fig. 4B), and the areas of Nanog-positive colonies were smaller than those of the control group (Fig. 4C and 4D). These results demonstrate that Activin A has a negative effect on EPI cell development and also affects the isolation and primary cloning of mouse ESCs.

### ESC and iPSC-secreted protein Activin A promotes TE lineage development

**Figure 5:**
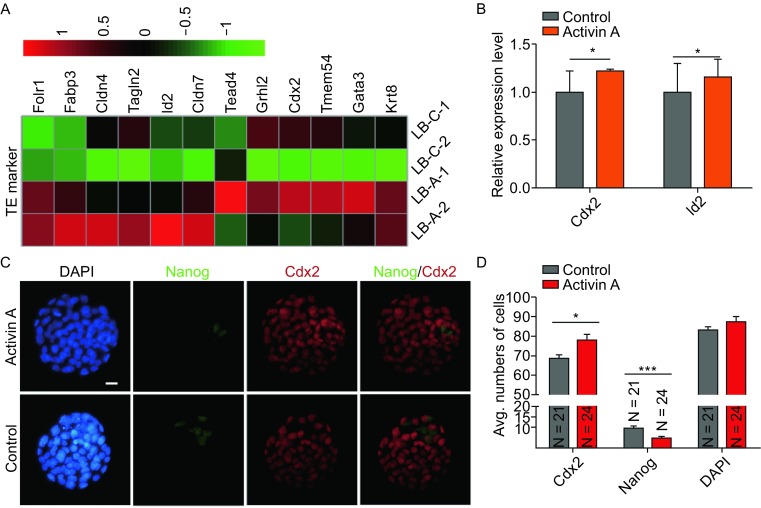
**Activin A promotes TE lineage**. (A) Heatmap of selected genes related to TE lineage. Heatmap was plotted with Z-score normalized RPKM. (B) Quantitative RT-PCR analyzed the expression of genes selected by heatmap of TE Markers. Error bars indicate SD (*n* = 3). *P < 0.05 by Student’s *t*-test. (C) Cdx2 immunostaining in Activin A-treated and untreated embryos at E4.5. Nuclei were stained with DAPI (Blue). Scale bars, 20 μm. (D) Average numbers of TE cells (Cdx2-positive cells) and EPI (Nanog-positive cells) in Activin A-treated and untreated embryos at E4.5. Error bars indicate SD. *P < 0.05, ***P < 0.001 by ANOVA. N is the number of embryos examined

There are two other lineages, TE and primitive endoderm (PE), in addition to the EPI lineage in the late blastocyst (Cockburn and Rossant, 2010; Wang and Dey, 2006). We hypothesized that the secreted protein Activin A might promote embryo blastomeres toward TE or PE lineages, and we further analyzed the RNA-Seq data. A heatmap showed that the expression levels of TE marker genes such as Id2 and Cdx2 were elevated in the Activin A group (Fig. 5A). These data were consistent with the quantitative RT-PCR results (Fig. 5B). Immunofluorescence staining also revealed that the TE marker protein CDX2 was clearly elevated in embryos following treatment with Activin A (Fig. 5C and 5D). These findings indicate that Activin A promotes TE lineage development and impedes EPI lineage development. However, our analysis data showed that Activin A has no obvious effects on PE lineage development.

### Increase in the contribution of ESCs to ICM and chimeras using Activin A-treated early blastocysts as recipients

**Figure 6:**
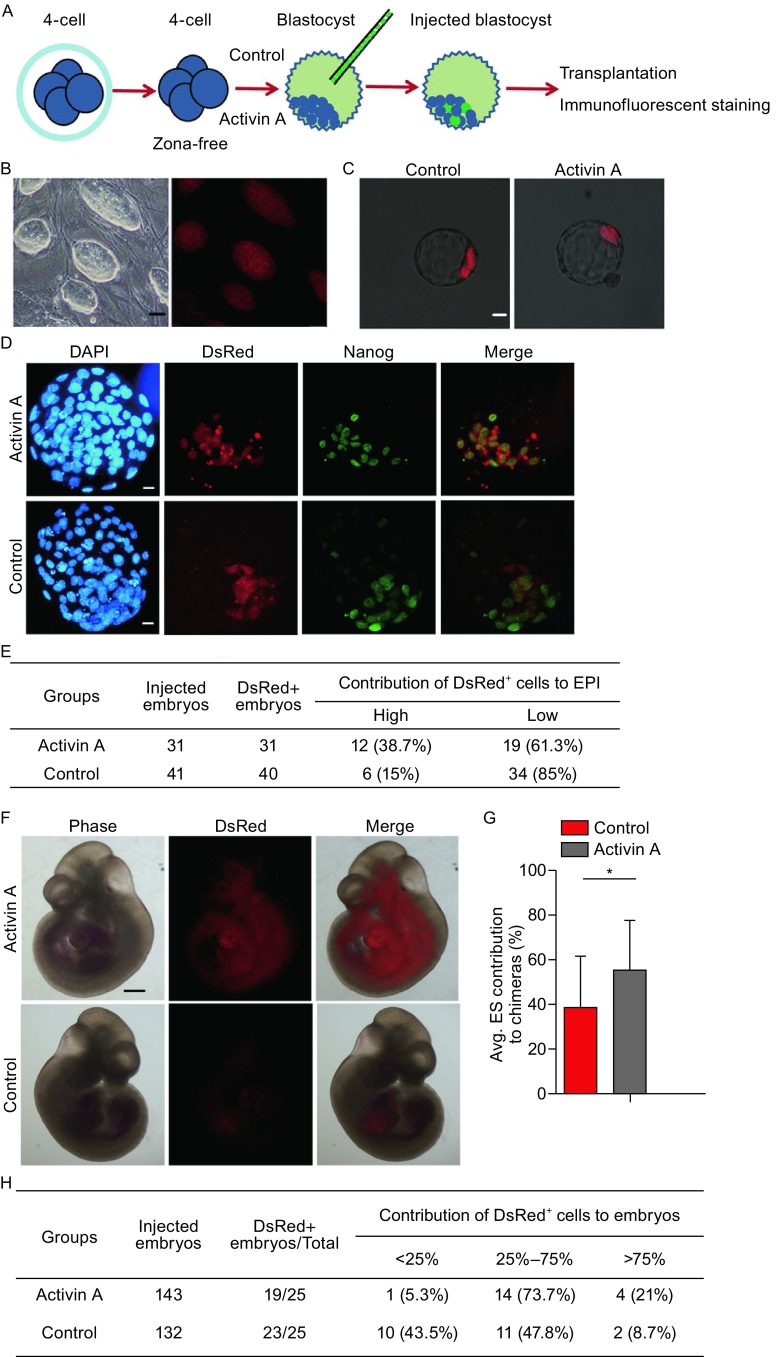
**Activin A enhances ESC contribution to chimeras generated by blastocyst injection**. (A) Experimental design. We treated 4-cell embryos without zona in KSOM including Activin A and then performed the blastocyst injection to test whether Activin A can affect the contribution of ESCs to chimeras. (B) The ESC colonies (left panel, bright field; right panel, DsRed). Scale bar, 20 μm. (C) ESC-injected embryos at E4.5. Scale bar, 20 µm. (D) Nanog immunostaining in E4.5 chimeric embryos after blastocyst injection. Nuclei were stained with DAPI (Blue). Scale bars, 20 μm. (E) Summary of ESC (DsRed) contribution to E4.5 embryos. *P* < 0.05 by Chi-square test. The high group indicates that DsRed ESCs almost overlaid Nanog-positive cells as shown in the top panel of Fig. 4D, and others are included in the low group. (F) Chimeric embryos at E10.5. Scale bar, 500 mm. (G) Average ESC contribution to chimeric embryos at E10.5 using ImageJ software. *P < 0.05 by ANOVA. (H) Summary of ESC (DsRed) contribution to E10.5 chimeric embryos. *P* < 0.05 by Chi-square test

We next performed the blastocyst injection assay to further examine the role of ESC-secreted proteins in chimeras producing. Activin A-treated blastocysts (cultured from 4-cell stage to early blastocyst at E3.5) were taken as host embryos, and G4-DsRed-MST ESCs were taken as donor cells (Fig. 6A and 6B). We detected the fates of ESCs that were injected into blastocysts when chimeric embryos developed to the late blastocyst stage. Fluorescent locations of ESCs and immunostaining of Nanog indicated that 38.7% of aggregated embryos showed a high contribution of ESCs to the EPI lineage in host embryos treated by Activin A compared with embryos in the control group (15%) (Fig. 6C–E). Additionally, we detected the E10.5 chimeric embryos after embryo transplantation, which showed that Activin A treatment increased the average contribution of ESCs to the chimeric fetus (Fig. 6F and 6G) and increased the rate of higher ESC-contributed chimeras (25%–75% and >75% ESCs contribution) while decreasing the rate of lower ESC-contributed chimeras (<25% ESCs contribution) (Fig. 6F and 6H). The results were similar to those in the E4.5 chimeras mentioned above. Taken together, the analysis and comparison of the ESC contribution in chimeric embryos at different stages demonstrate that the ESC-secreted protein Activin A following treatment for host embryos can increase the contribution of ESCs to the chimeras produced by blastocyst injection by impeding EPI lineage development of the host embryos.

## DISCUSSION

The injection of ESCs into 4-cell stage embryos resulted in higher ESC contributions to the chimera than the blastocyst-based approach (Huang et al., 2008; Poueymirou et al., 2007). One possible reason for this difference is that exogenous ESCs have some effects on early embryo development. As reported, ESCs decrease the cell numbers of the ICM of host embryo-origin during blastocyst formation (Humiecka et al., 2016). However, there are different views about how ESCs exert regulatory roles in mouse early embryo development. One theory is that ESCs influence blastomere division (Humiecka et al., 2016), while another is that ESCs have a competitive advantage compared with early blastomeres (Poueymirou et al., 2007).

At the onset of our study, we hypothesized that ESCs or iPSCs influence the development of early embryos by secreting certain factors. We confirmed that F0 ESC or iPSC-derived mice could be generated by the injection of approximately 10 cells into a 4-cell stage embryo. A previous study reported that the injection of more than 9 cells through the laser-assisted injection system has no added benefit because the excess cells tend to leak out through the opening in the zona pellucida (Poueymirou et al., 2007). Using the Piezo Micro Manipulator (PMM-150), we can easily inject 20 cells into one embryo, and we found that the injection of 20 iPSCs or ESCs instead of 10 cells into embryos increased the probability of obtaining F0 generation ES-mice or iPS-mice. We then explored the evidence supporting that ESC and iPSC-secreted proteins can improve their EPI competency after injection into recipient embryos. The key protein, Activin A, plays a positive role in generating chimeras with a higher degree of ESC contribution via impeding the EPI lineage development of host embryos. This can be explained by the results that numbers of EPI cells decreased during the formation of the blastocysts cultured with the condition medium from ESCs and iPSCs or cultured with Activin A (Figs. 2 and 3). These results indicated that the ESC or iPSC-secreted protein Activin A hinders EPI lineage development. Because of the small number of EPI cells, a high-ratio chimera could be generated after injection of the same number of pluripotent stem cells into recipient embryos, which increased the ratio of exogenous cells in the ICM. This conclusion was also supported by the transcriptome of the blastocysts treated with Activin A from the 4-cell stage (Fig. 3). Transcription factors such as Pou5f1 and Nanog, which are key regulators during EPI specification, were significantly reduced after treatment with Activin A. A previous report, which supports our conclusion, showed that Activin A can hinder EPI formation in early mouse embryos (Ghimire et al., 2015). These associations were further confirmed by our result that the cell number of EPI increased in the presence of the Activin receptor inhibitor SB431542 (Fig. S3). These data clearly demonstrate that ESC-secreted proteins facilitate the production of a higher ESC contribution to chimeras by impeding the development of EPI cells in host embryos.

We further revealed that Activin A increased the numbers of the TE lineage (Fig. 5), which may provide clues to explain the effects of Activin A or even TGF-β signaling pathways on the preimplantation embryonic development. Nodal/Activin signaling has been shown to be required for trophoblast stem cell (TSC) renewal in culture conditions, while working with other factors (Guzman-Ayala et al., 2004; Ohinata and Tsukiyama, 2014). This result can explain why the TE or TSC marker gene expression level increased in the blastocysts treated with Activin A, while the PE-related genes remain comparable. Using Activin A-treated embryos as hosts enhances the ESC contribution to the chimeras produced by the traditional blastocyst-based approach. This result further confirmed the role of Activin A in preimplantation embryos, as well as in the 4-cell embryo injection assay. This process will have great potential to efficiently produce gene-edited mice.

In summary, we conclude that the ESC and iPSC secrete protein Activin A to improve their EPI competency after injection into recipient embryos through impacting on the development of early embryo. Our study not only gives an effective explanation for the higher contribution of pluripotent stem cells to the EPI component and chimeras produced by the 4-cell approach compared with blastocyst injection, but also provides a new idea and theoretical basis for the optimization of chimera production systems in the future.

## MATERIALS AND METHODS

### Animal experiments

All animal studies proceeded according to the guidelines of the Institute Animal Care and Use Committee and were approved by the Animal Care and Use Committee of China Agricultural University. We used CD1 (ICR) mice as the embryo donors and recipients, which were purchased from Beijing Vital River Laboratory Animal Technology Co., Ltd. (Beijing, China). All mice were maintained in specific pathogen-free (SPF) conditions with a 12-h dark/12-h light cycle.

### Cell culture

The ESCs and iPSCs were maintained on mitomycin C-treated mouse embryonic fibroblast (MEF) feeder cells in the ESC medium. The ESC medium contained DMEM (Invitrogen) with 15% FBS, 2 mmol/L GlutaMAX, 1 mmol/L sodium pyruvate, 2 mmol/L nonessential amino acids, 0.1 mmol/L 2-mercaptophenol (all from Gibco), and 1000 units/mL LIF (Millipore).

### Condition medium collection and mass spectroscopy

The initial cells (ESCs and iPSCs) were seeded at a density of 5.0 × 10^5^ cells per well of a 6-well plate on feeder cells. The original medium was replaced with serum-free fresh ESC medium 12 h later. The supernatant was collected and then centrifuged at 4,000 rpm for 60 min at 4°C after 12 h. The supernatant was stored at −80°C before further processing, such as mass spectrometry.

Liquid chromatography-tandem mass spectrometry (LC-MS/MS) analysis was carried out by Capitbalbio Technology using the Q Exactive mass spectrometer (Thermo Scientific, CA). Mass spectrometry analysis was performed in a data-dependent manner, with full scans (350–1,600 m/z) acquired using an Orbitrap mass analyzer at a mass resolution of 70,000 at 400 m/z in Q Exactive.

### Embryo collection and culture *in vitro*

To obtain 2-cell stage embryos, female mice were superovulated by intraperitoneal injection of 5 international units (IU) of PMSG, followed by 5 IU HCG 46–48 h later, and mated with male mice. 2-cell stage embryos were obtained by flushing the oviduct with M2 at E1.5. Embryos were washed in M2 (Millipore) and then transferred into 10 μL KSOM (Millipore) drops covered with mineral oil (Sigma) on a tissue culture dish. The embryos were maintained at 37°C with 5% CO_2_ in an incubator (Thermo Scientific).

For SB431542-treated embryos, 4-cell embryos were cultured in KSOM with 10 μmol/L SB431542, and embryos in the control group were cultured with an equivalent amount of DMSO in KSOM.

For condition medium-treated embryos, the zonas of 4-cell embryos were removed by acidic Tyrode’s solution (Millipore). Zona-free 4-cell embryos were cultured in the mixed medium including 50% KSOM and 50% condition medium from ESCs and iPSCs. Zona-free embryos at the 4-cell stage in the control group were cultured in the mixed medium, including 50% KSOM and 50% condition medium from feeder cells. Embryos were fixed at E4.5 for EPI detection.

For Activin A-treated embryos, zona-free 4-cell embryos were cultured in KSOM with 500 ng/mL Activin A. Zona-free embryos at the 4-cell stage in the control group were cultured with an equivalent volume of 0.1% BSA in KSOM. Embryos were fixed at E4.5 for EPI detection.

For outgrowth assays, embryos were cultured in KSOM with Activin A as above until E4.5 and then transferred on feeder cells in the ESC medium plus CHIR99021 and PD0325901 (Selleck). After treating for 8 days, outgrowths were fixed for immunofluorescence staining.

### Immunofluorescence staining

Embryos were fixed with 4% paraformaldehyde in DPBS for 30 min at room temperature and then washed three times with 0.2% BSA in DPBS. Embryos were then permeabilized at room temperature in 0.5% Triton X-100 in DPBS for 30 min. After washing according to the above method, embryos were incubated with primary antibodies in 0.2% BSA for 4 h at room temperature, washed with 0.2% BSA and incubated with secondary antibodies in 0.2% BSA for 1 h at room temperature. Following washing with 0.2% BSA, the embryos were incubated for 3 min in the mounting medium with DAPI.

Immunostaining was performed with Nanog (Cell Signaling Technology, 1:500) and Cdx2 primary antibodies (Biogenex, 1:200). The secondary antibodies used in this research were as follows: Alexa594 goat anti-rabbit IgG, Alexa488 goat anti-rabbit IgG, and Alexa594 donkey anti-mouse IgG antibodies (Invitrogen, 1:500).

### ESC or iPSC injection and embryo transfer

The mouse ESCs and iPSCs were introduced into early embryos by Piezo micromanipulation as previously described (Huang et al., 2008; Kawase et al., 2001). For the generation of F0 iPSC-derived mice, 10 or 20 iPSCs were injected into 4-cell stage embryos. For ESC contribution assays, 15 ESCs were injected into the blastocyst treated by Activin A as described above. The same numbers of ESCs were injected into embryos in the control group.

CD1 females mated with vasectomized CD1 males were used as pseudopregnant mice. Embryos with injected ESCs were transferred into the uterus or oviduct of pseudopregnant mice, depending on the developmental stage. Blastocysts were transferred into the uterus of pseudopregnant females at 2.5 days post coitum (dpc). Embryos at the morula stage were transferred into the oviduct of 0.5 dpc recipients. We transferred 16–20 embryos per recipient.

## ACCESSION NUMBERS

Raw reads of mouse blastocyst transcriptome data have been submitted to the NCBI Sequence Read Archive (SRA; http://www.ncbi.nlm.nih.gov/sra/) under accession number SRA561196.

## Electronic supplementary material

Below is the link to the electronic supplementary material.

Supplementary material 1 (PDF 518 kb)
