# Supplementary material for: Pluripotent stem cells secrete Activin A to improve their epiblast competency after injection into recipient embryos
Source: Protein Cell. 2017 Oct 12;9(8):717–28. doi: 10.1007/s13238-017-0470-y (PMC6053354; doi:10.1007/s13238-017-0470-y)
Supplement: Supplementary file 1 — Supplementary material 1 (PDF 518 kb) [file 13238_2017_470_MOESM1_ESM.pdf]

Supplemental Figure Legends

Figure S1. Donor ESCs impede EPI development of host embryos. Related to Figure 1.

(A) Schematic of method used for 4-cell embryo injection.

(B) Blastocyst generated by 4-cell stage embryo injection. Scale bar, 20 µm.

(C) Nanog immunostaining in E4.5 embryos from 4-cell embryo injection. Nuclei were stained with DAPI (Blue). Scale bar, 20 µm.

(D) Summary of donor ES cell contribution to E4.5 embryos generated by 4-cell stage embryo injection. The embryos were divided into 3 groups, containing blastocysts with ES cell-derived, chimeric and host-derived EPI. $p<0.05$ by Chi-square test.

Figure S2. The expression of Activin A receptors in mouse early embryos. Related to Figure 3.

The boxplot was plotted in the type of Tukey boxplot, the whisker of the boxplot are always the 25th and 75th percentile (the lower and upper quartiles, respectively), and the band near the middle of the box is always the 50th percentile (the median).

Figure S3. TGF-β inhibitor SB431542 increases cell numbers of EPI lineage. Related to Figure 3.

(A) Nanog immunostaining in SB431542 treated and untreated embryos at E4.5. Nuclei were stained with DAPI (Blue). Scale bars, 20 µm.

(B) Average numbers of EPI cells (Nanog expressing cells) in SB431542 treated and untreated embryos at E4.5. Error bars indicate SD. *$p<0.05$ by ANOVA. N is the number of embryos examined.

Table S1. Summary of microsatellite analysis of tissues from iPS cell-derived mice using D12Mit60 primers. Related to Figure 1.

Figure S1

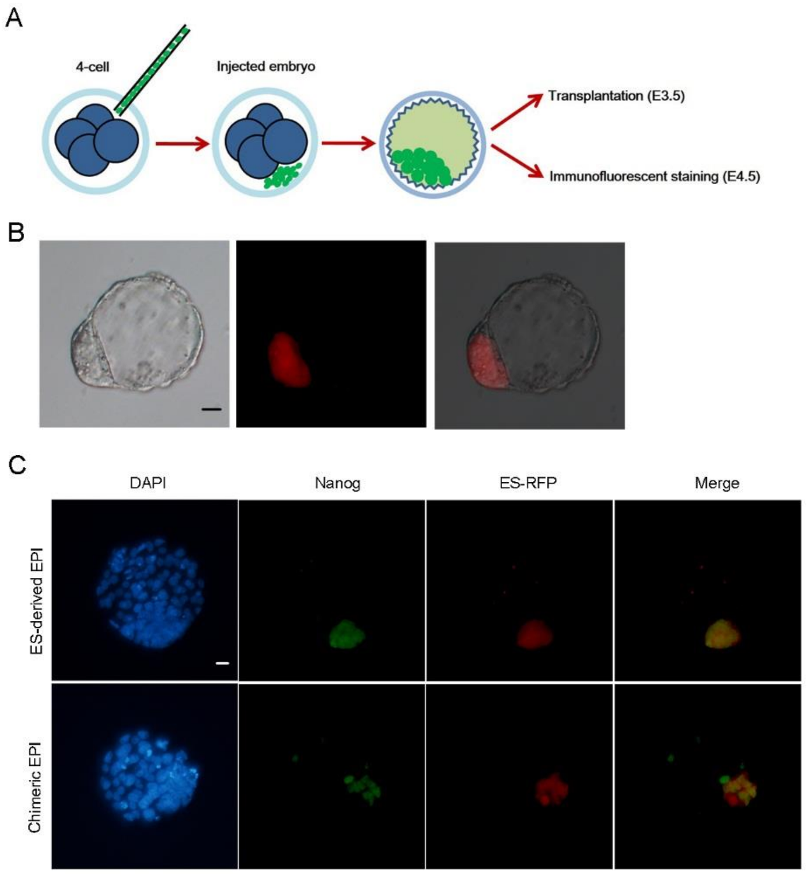

D

Groups	Blastocysts with ES-derived EPI	Blastocysts with chimeric EPI	Blastocysts with host-derived EPI
10 cells / embryo	5 (31.25%)	7 (43.75%)	4 (25%)
20 cells / embryo	21 (75%)	4 (14.3%)	3 (10.7%)

Figure S2

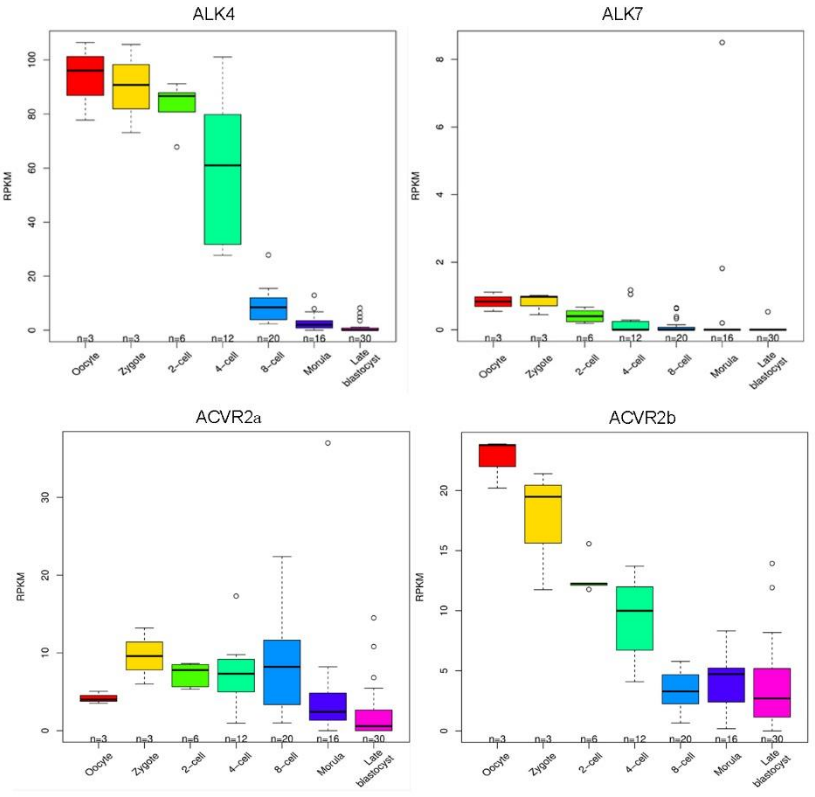
ALK4
ALK7
ACVR2a
ACVR2b
RPKM
n=3
n=3
n=6
n=12
n=20
n=16
n=30
Oocyte
Zygote
2-cell
4-cell
8-cell
Morula
Late blastocyst

Figure S3

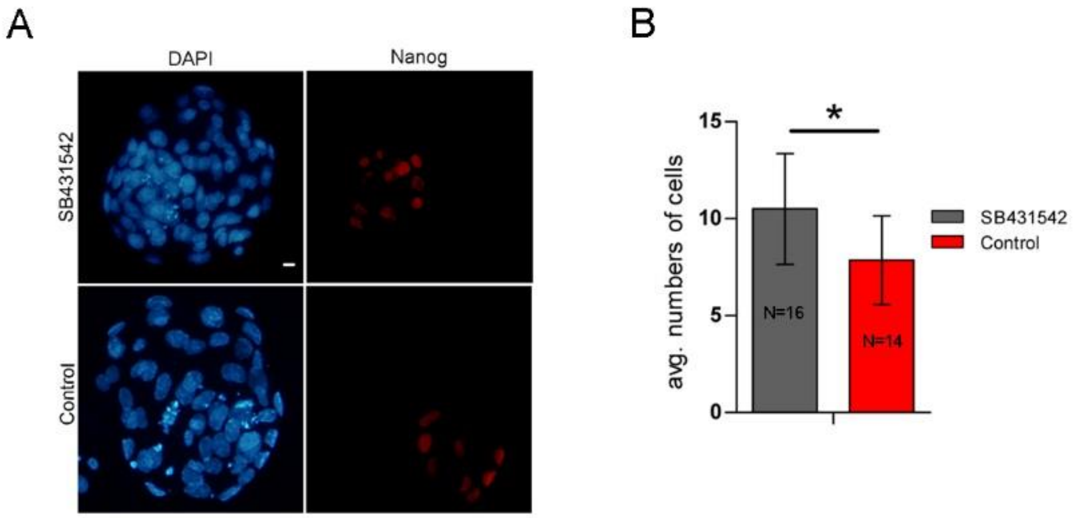
A
DAPI
Nanog
SB431542
Control
B
*
15
10
5
0
avg. numbers of cells
N=16
N=14
SB431542
Control

Table S1. Summary of microsatellite analysis of tissues from iPS cell-derived mice using D12Mit60 primers. Microsatellite PCR products were separated on an ABI 3730xl DNA analyser and alleles were sized against the size standard GS-500 using GenMapper software (Applied Biosystems)

	Sample Name	Allele 1	Allele 2
Fully iPS-mice-1 from iPS cell line SKR B11	heart	128	144
	lung	128	144
	testis	128	144
	spleen	128	144
	kidney	128	144
	liver	128	144
	muscle	128	144
	limb	128	144
	tail	128	144
	brain	128	144
Fully iPS-mice-2 from iPS cell line SKR B11	limb	128	144
	tail	128	144
	brain	128	144
	lung	128	144
	skin	128	144
	liver	128	144
	spleen	128	144
	heart	128	144
	stamock	128	144
	kidney	128	144
Embryo donor mice	CD1	137	137
iPS cell line from C57x129 MEF	SKR B11	128	144

Supplemental Experimental Procedures

KEY RESOURCES TABLE

Reagent or Resource	Source
Cell lines	
G4-DsRed-MST-ES cell	Mount Sinai Hospital, Canada(Vintersten et al., 2004)
R1-ES cell	Mount Sinai Hospital, Canada
OSKT-iPS cell	(Han et al., 2010)
SKR-iPS cell	(Heng et al., 2010)
SKRC-iPS cell	(Heng et al., 2010)
Primers for quantitative RT-PCR (5' to 3')	
Sox2-F: AAGGGTTCTTGCTGGGTTTT	(Carter et al., 2014)
Sox2-R: AGACCACGAAAACGGTCTTG	
Id2-F: CTATCGTCAGCCTGCATCAC	(Choi et al., 2009)
Id2-R: ATTCAGATGCCTGCAAGGAC	
β-actin-F: GATCTGGCACCACACCTTCT	(Valenta et al., 2006)
β-actin-R: GGGGTGTTGAAGGTCTCAAA	
Cdx2-F: AGAGGAAGGGCGAGGAGA	(He et al., 2013)
Cdx2-R: CCAGCTCACTTTTCCTCCTG	
Pou5f1-F: GTTGGAGAAGGTGGAACCAA	(Rudloff and Kemler, 2012)
Pou5f1-R: CTCCTTCTGCAGGGCTTTC	
Nanog-F: TTCTTGCTTACAAGGGTCTGC	(Rudloff and Kemler, 2012)

Nanog-R: AGAGGAAGGGCGAGGAGA

RNA-seq

Direct cDNA synthesis was performed from intact cells using the SMART-Seq v4 Ultra Low Input RNA Kit (Clontech) for Sequencing according to the manufacturer's instructions. Briefly, cDNA libraries were created using the NEBNext® Ultra™ DNA Library Prep Kit (NEB) for Illumina following the manufacturer's instructions. The libraries were sequenced on the HiSeq 2500 sequencing platform (Illumina).

Bioinformatic analysis of RNA-seq

Short reads were mapped to the mouse mm10 reference genome with HISAT2 (Kim et al., 2015). The annotation files were obtained from the Ensembl database: mouse (GRCm38.78). The aligned reads of each sample were assembled with cufflinks (version 2.21) (Trapnell et al., 2012), and we performed Reference Annotation-Based Transcript (RABT) assembly (Roberts et al., 2011) with the Ensembl annotation to compensate incompletely assembled transcripts caused by read coverage gaps in the regions of known genes in the assembly. We used HTseq (Anders et al., 2015) to obtain read count tables from the binary sequence alignment (BAM) file. The DEseq2 (Love et al., 2014) package was used to normalize count data and to calculate the differential expression tests between different embryonic stages. A gene will be reported as a significant differential expression gene if the test indicates that the FDR-adjusted p-value was less than 0.05 and the $\log_2$ fold change was greater than 1.

PCR and Microsatellite Analysis

DNA samples were obtained from iPS-mice. Mouse microsatellites (D12Mit60) and corresponding primer pairs have been previously described (Copeland et al., 1993). Genomic

DNA (30 ng) was amplified by 35 cycles of fluorescent PCR using the Platinum Taq DNA Polymerase system (Invitrogen). Microsatellite PCR products were separated on an ABI 3730*xl* DNA analyzer, and alleles were sized against the size standard GS-500 using GeneMapper software (Applied Biosystems).

Quantitative RT-PCR

Total RNA was isolated from embryos using the Pico-Pure RNA Isolation Kit (Applied Biosystems) and prepared for reverse transcription using M-MLV Reverse Transcriptase (Promega). The total RNA was extracted from eight embryos in each group. The cDNAs were then used for quantitative RT-PCR with SYBR Green (Roche). The data were analyzed by the comparative CT ($2^{-\Delta\Delta CT}$) method. The ΔCT was calculated using β-actin as an internal control. Reactions were carried out in triplicate using the following primers: Nanog-F: 5′-TTCTTGCTTACAAGGGTCTGC-3′ and Nanog-R: 5′-AGAGGAAGGGCGAGGAGA-3′; Pou5f1-F: 5′- GTTGGAGAAGGTGGAACCAA-3′ and Pou5f1-R: 5′-CTCCTTCTGCAGGGCTTTC-3′; Cdx2-F: 5′-AGAGGAAGGGCGAGGAGA-3′ and Cdx2-R: 5′-CCAGCTCACTTTTCCTCCTG-3′; Sox2-F: 5′-AAGGGTTCTTGCTGGGTTTT-3′ and Sox2-R: 5′-AGACCACGAAAACGGTCTTG-3′; Id2-F: 5′-CTATCGTCAGCCTGCATCAC-3′ and R:5′-ATTCAGATGCCTGCAAGGAC-3′; β-actin-F: 5′-GATCTGGCACCACACCTTCT-3′ and β-actin-R: 5′-GGGGTGTTGAAGGTCTCAAA-3′

Quantification and Statistical Analysis

Statistical analysis was performed using IBM SPSS Statistics 21 software. Image J was used to predetermine sample areas. We tested for differences between samples using the chi-square test or ANOVA. Error bars represent SD unless otherwise indicated. Please refer to figures and figure legends for the numbers of embryos used per experiment.

Supplemental References

Anders, S., Pyl, P.T., and Huber, W. (2015). HTSeq--a Python framework to work with high-throughput sequencing data. Bioinformatics 31, 166-169.

Carter, A.C., Davis-Dusenbery, B.N., Koszka, K., Ichida, J.K., and Eggan, K. (2014). Nanog-independent reprogramming to iPSCs with canonical factors. Stem cell reports 2, 119-126.

Choi, Y.S., Chakrabarti, R., Escamilla-Hernandez, R., and Sinha, S. (2009). Elf5 conditional knockout mice reveal its role as a master regulator in mammary alveolar development: failure of Stat5 activation and functional differentiation in the absence of Elf5. Dev Biol 329, 227-241.

Copeland, N.G., Jenkins, N.A., Gilbert, D.J., Eppig, J.T., Maltais, L.J., Miller, J.C., Dietrich, W.F., Weaver, A., Lincoln, S.E., Steen, R.G., et al. (1993). A genetic linkage map of the mouse: current applications and future prospects. Science 262, 57-66.

Han, J., Yuan, P., Yang, H., Zhang, J., Soh, B.S., Li, P., Lim, S.L., Cao, S., Tay, J., Orlov, Y.L., et al. (2010). Tbx3 improves the germ-line competency of induced pluripotent stem cells. Nature 463, 1096-1100.

He, J., Shen, L., Wan, M., Taranova, O., Wu, H., and Zhang, Y. (2013). Kdm2b maintains murine embryonic stem cell status by recruiting PRC1 complex to CpG islands of developmental genes. Nature cell biology 15, 373-384.

Heng, J.C.D., Feng, B., Han, J.Y., Jiang, J.M., Kraus, P., Ng, J.H., Orlov, Y.L., Huss, M., Yang, L., Lufkin, T., et al. (2010). The Nuclear Receptor Nr5a2 Can Replace Oct4 in the Reprogramming of Murine Somatic Cells to Pluripotent Cells. Cell Stem Cell 6, 167-174.

Kim, D., Langmead, B., and Salzberg, S.L. (2015). HISAT: a fast spliced aligner with low memory requirements. Nat Methods 12, 357-360.

Love, M.I., Huber, W., and Anders, S. (2014). Moderated estimation of fold change and dispersion for RNA-seq data with DESeq2. Genome Biol 15, 550.

Roberts, A., Pimentel, H., Trapnell, C., and Pachter, L. (2011). Identification of novel transcripts in annotated genomes using RNA-Seq. Bioinformatics 27, 2325-2329.

Rudloff, S., and Kemler, R. (2012). Differential requirements for beta-catenin during mouse development. Development 139, 3711-3721.

Trapnell, C., Roberts, A., Goff, L., Pertea, G., Kim, D., Kelley, D.R., Pimentel, H., Salzberg, S.L., Rinn, J.L.,

and Pachter, L. (2012). Differential gene and transcript expression analysis of RNA-seq experiments with TopHat and Cufflinks. Nat Protoc 7, 562-578.

Valenta, T., Lukas, J., Doubravska, L., Fafilek, B., and Korinek, V. (2006). HIC1 attenuates Wnt signaling by recruitment of TCF-4 and beta-catenin to the nuclear bodies. EMBO J 25, 2326-2337.

Vintersten, K., Monetti, C., Gertsenstein, M., Zhang, P., Laszlo, L., Biechele, S., and Nagy, A. (2004). Mouse in red: red fluorescent protein expression in mouse ES cells, embryos, and adult animals. Genesis 40, 241-246.
